# The potential role of herbal extract Wedelolactone for treating particle-induced osteolysis: an in vivo study

**DOI:** 10.1186/s13018-022-03228-9

**Published:** 2022-06-28

**Authors:** Yung-Chang Lu, Ting-Kuo Chang, Tzu-Chiao Lin, Shu-Ting Yeh, Hsu-Wei Fang, Chun-Hsiung Huang, Chang-Hung Huang

**Affiliations:** 1 Department of Medicine, MacKay Medical College, New Taipei City, Taiwan; 2 Department of Medical Research, MacKay Memorial Hospital, New Taipei City, Taiwan; 3 Department of Orthopaedic Surgery, MacKay Memorial Hospital, Taipei, Taiwan; 4 Department of Chemical Engineering and Biotechnology, National Taipei University of Technology, Taipei, Taiwan; 5 Department of Orthopaedic Surgery, Changhua Christian Hospital, Changhua, Taiwan; 6 School of Dentistry, National Yang Ming Chiao Tung University, Taipei, Taiwan; 7 Institute of Geriatric Welfare Technology and Science, MacKay Medical College, New Taipei City, Taiwan

**Keywords:** Particle-induced osteolysis, Wedelolactone, Herbal medicine, Murine calvarial model

## Abstract

**Background:**

Osteolysis is one of the most prevalent clinical complications affecting people who undergo total joint replacement (TJR). Wedelolactone (WDL) is a coumestan compound derived from the *Wedelia chinensis* plant and has been demonstrated to exhibit anti-inflammatory properties. This study aimed to investigate the oral administration of WDL as a potential treatment for particle-induced osteolysis using a well-established mice calvarial disease model.

**Methods:**

Thirty-two C57BL/6 J mice were randomized into four groups: Sham, vehicle, osteolysis group with oral WDL treatment for 4 weeks (WDL 4w), and osteolysis group treated for 8 weeks (WDL 8w). Micro-CT was used to quantitatively analyze the bone mineral density (BMD), bone volume/tissue volume (BV/TV) and trabecular bone thickness (Tb.Th). Osteoclast numbers were also measured from histological slides by two investigators who were blind to the treatment used.

**Results:**

The results from micro-CT observation showed that BMD in the WDL 8w group improved significantly over the vehicle group (*p* < 0.05), but there was no significant difference between WDL 4w and 8w for BV/TV and Tb.Th. Osteoclast numbers in the WDL 4w group were also lower than the vehicle group (*p* < 0.05), but the difference between WDL 8w and 4w groups was not significant.

**Conclusions:**

Particle-induced osteolysis is an inevitable long-term complication after TJR. The results of this animal study indicate that an oral administration of WDL can help reduce the severity of osteolysis without adverse effects.

## Background

Artificial joint replacement (AJR) is considered an effective method of treating severe joint degeneration [1]. However, periprosthetic osteolysis resulting from the deposition of wear particles from the articulating joint is one of the major clinical complications following AJR [2]. Wear particles can stimulate inflammatory responses and osteoclastic resorption processes at the bone implant interface, which consequently often leads to implant loosening [3]. Although bearing materials have been introduced to reduce the generation of wear particles, osteolysis is still prevalent and is considered a major long-term complication of AJR.

Besides changing the base material of the implant, pharmaceuticals have also been investigated as a method for reducing osteolysis [4]. Bisphosphonates are well-known drugs for treating osteoporosis, but have also been shown to be effective at suppressing osteolysis [5, 6]. Similarly, statins, which are lipid-lowering agents, have been reported to reduce particle-induced osteolysis in a murine calvarial model [5]. However, such drugs can also introduce considerable serious side effects, such as atypical femoral bone fracture and osteonecrosis of the jaw [7–9].

Animal models are often used for investigating mechanisms that can lead to particle-induced osteolysis and for evaluating suitable treatment methods [5, 10–14]. A previous study by our institute investigated whether strontium ranelate (SR) [11], a drug for osteoporosis, could be effectively used to combat osteolysis. After gavage-feeding mice for up to 4 weeks, the results showed a significant increase in bone mineral density (BMD), bone volume/tissue volume (BV/TV) and trabecular thickness (Tb.Th), and a significant reduction in osteoclast numbers [11]. However, long-term use of SR may increase the risk of cardiovascular disease [15–17].

As an alternative to pharmaceuticals, Chinese herbal medicines are considered a more natural solution with fewer side effects [18]. The International Organization for Standardization has also assembled a technical committee (ISO/TC 249) to standardize medical fields derived from Chinese medicine. With the increasing popularity, more resources and attention have been given to the potential benefits for treating disease [19–21].

Wedelolactone (WDL) is a coumestan compound extracted from the *Wedelia Chinensis* plant [22]. WDL is considered a traditional Chinese herbal medicine with strong anti-inflammatory properties [23–27]. Recent studies have shown that WDL can promote hair growth [28] and is hepatoprotective [29, 30], neuroprotective [31] and anti-carcinogenic [32, 33]. However, few studies have reported on the potential of WDL for treating diseases of the skeletal system. Based on the ability of WDL to reduce inflammation [23–27] and inhibit osteoclastogenesis [34, 35], it is hypothesized that WDL could also play a role in reducing the risk of particle-induced osteolysis. This study used a well-established murine calvarial osteolysis model to investigate whether WDL administered orally could reduce the severity of osteolysis.

## Methods

### Establishing the calvarial particle-induced osteolysis model

**Fig. 1:**
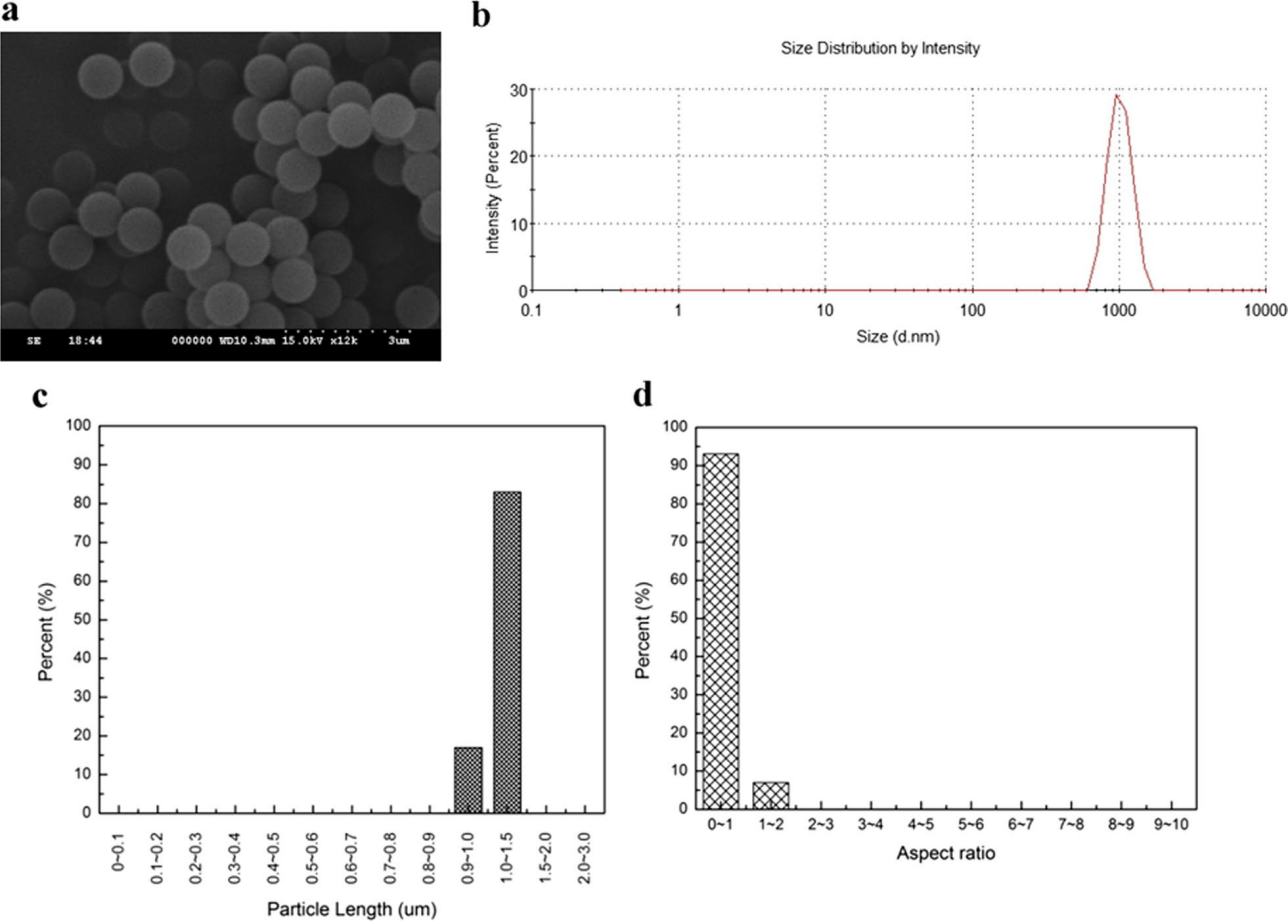
SEM image (× 12,000) of polystyrene (PS) particles (**a**). The particle size distribution by light-scattering analysis (**b**). The particle size distribution (**c**) and the aspect ratio (**d**) of the particles from SEM images

The protocol for this experiment was approved by the Institutional Animal Care and Use Committee at the institute where the study was performed. Thirty-two 6-week-old C57BL/6 J female mice were supplied by BioLASCO (Taipei, Taiwan), an AAALAC certified biotechnology company. The animals were kept in a room at 24℃, 50% humidity, and with a 12 h light/dark cycle (light from AM 7:00 to PM 7:00). The animals were randomly separated into four groups: (1) sham group (*n* = 8) (underwent surgery only) (2) vehicle group (*n* = 8) (implanted with PS particles) [36–38], (3) WDL 4w (*n* = 8, implanted with PS particles and treated with WDL for 4 weeks. 7 animals remained at the end of the experiment) and (4) WDL 8w (*n* = 8, treatment for 8 weeks). The vehicle group and WDL-treated groups were injected with 1 mg PS particles/100 μl HA [10, 25]. The polystyrene particles (Polystyrene Latex Spheres, 610–38) were purchased from TED PELLA, Inc. (CA, USA). Three hundred particles were randomly selected and SEM was used to measure the particle size and aspect ratio (Fig. 1a). The particles were found to be 1.03 ± 0.04 μm and 0.99 ± 0.03, respectively (Fig. 1b, c). The particles were also confirmed to have an endotoxin level below 0.25 EU/mL using a Limulus Ambocyte Lysate assay kit (ToxinSensor™ gel clot endotoxin assay kit, GenScript, NJ, USA) and then suspended in hyaluronic acid.

To inject the particles, the mice were anesthetized with 100 mg/kg of Zoletil 50 and 10 mg/kg Rompun by intraperitoneal injection. A 0.5 × 0.5 cm area of the middle calvaria was exposed by sagittal incision. After removing the periosteum intact, the particle suspension was spread over the area and the incision was closed with sutures. After 2 week post-surgery, the WDL 4w and 8w groups were gavage-fed with Wedelolactone (Y0001599, European Pharmacopoeia Reference Standard, Sigma-Aldrich, USA) at a dose of 4 mg/kg/day for 5 days/week [21]. WDL was dissolved by DMSO as stock solution and diluted with phosphate-buffered saline (PBS) into 10 volumes to attain a dosage of 4 mg/kg. The vehicle group was gavage-fed the vehicle solution (10% DMSO in PBS). The sham group, vehicle group and WDL 4w group were then sacrificed after 4 weeks of feeding either the vehicle or WDL, and the WDL 8w group was sacrificed after 8 weeks of feeding WDL.

### Micro-CT imaging analysis

The calvarias were fixed in 10% buffered formalin for 24 h, and then transferred to 70% ethanol for 24 h. The specimens were scanned with the micro-CT system Skyscan 1076 (Bruker micro-CT, Kontich, Belgium) at a resolution of 2048 × 2048. Three-dimensional images were reconstructed in Skyscan with a voxel size of 9 μm [10, 25]. A spherical volume of interest (VOI) with a diameter of 5 mm was then defined with the bregma as the center. Within this VOI, the bone mineral density (BMD, mg/cc), the ratio of bone volume to tissue volume (BV/TV, %) and trabecular thickness (Tb.Th) were recorded for each group.

### Histological analysis

The calvarias were decalcificated in 10% ethylenediaminetetraacetic acid (EDTA) for 2 weeks, and then embedded in paraffin. Each section with a thickness of 5 μm were taken in the sagittal plane centered over the particle-treated area. The sections were then stained with hematoxylin and eosin (H&E stain) to observe the morphology of cellular inflammatory responses from the connective tissue. A tartrate-resistant acid phosphatase (TRAP) stain was performed using a commercial TRAP kit (#386A, Sigma-Aldrich). The number of osteoclasts was determined by counting the number of TRAP-positive multinucleated cells by two coauthors to eliminate intra- and inter-observer error.

### Statistical analysis

The data was analyzed using one-way analysis of variance (ANOVA) to show the difference between groups. Multiple comparisons were adjusted with a Bonferroni post hoc test. Results were reported as mean ± standard deviation (SD). Any *p* value less than 0.05 was considered significantly different.

## Results

### Micro-CT imaging analysis

**Fig. 2:**
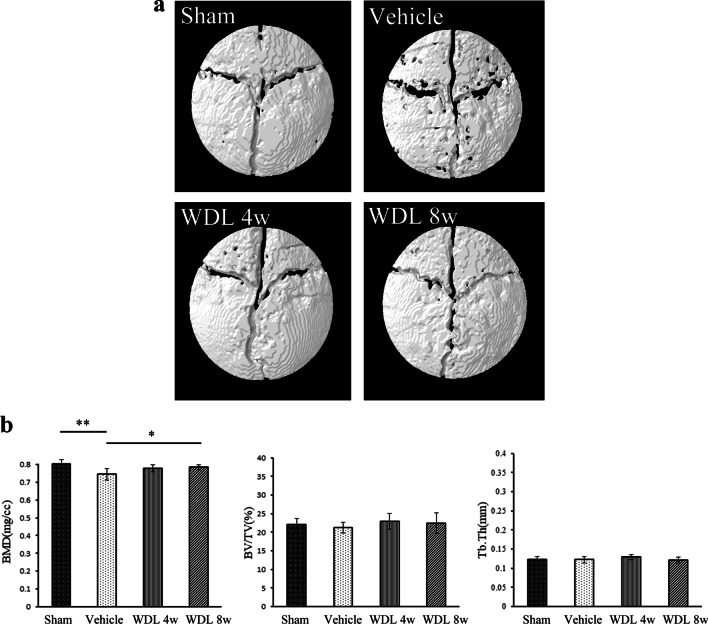
Reconstructed image of the VOI with the bregma at the center. The VOI is defined with a diameter of 5 mm (**a**). Micro-CT image of bone formation in a particle-induced osteolysis model measured at 4 and 8 weeks after feeding WDL (**b**) (*p < 0.05; ** *p* < 0.01, as determined using ANOVA testing)

A visual analysis of the three dimensional (3D) reconstructed micro-CT images showed clear differences between the sham group, vehicle group, and WDL groups (Fig. 2a). The images showed typical osteolysis with pores in the sham group, but both the size and number of pores decreased in WDL-treated groups. The presence of PS particles significantly decreased the BMD in the vehicle group by 7.8% when compared to the sham group (0.74 ± 0.03 for vehicle group and 0.801 ± 0.03 for sham group, *p* < 0.01). Both WDL groups showed an increase in BMD (Fig. 2b), with the 8w group showing a significant increase of 5.1% in comparison to the vehicle group (0.78 ± 0.01 for 8w group and 0.74 ± 0.03 for vehicle group, *p* < 0.05). The BV/TV in the vehicle group decreased by 4.1% in comparison to the sham group (21.23 ± 1.43 in vehicle group versus 22.1 ± 1.54 in sham group) but increased in the WDL 4w group (22.96 ± 2.07) and WDL 8w group (22.44 ± 2.78). However, there were no significant differences in BV/TV and Tb.Th between the WDL 4w and WDL 8w groups (Fig. 2b).

### Histomorphometric analysis

**Fig. 3:**
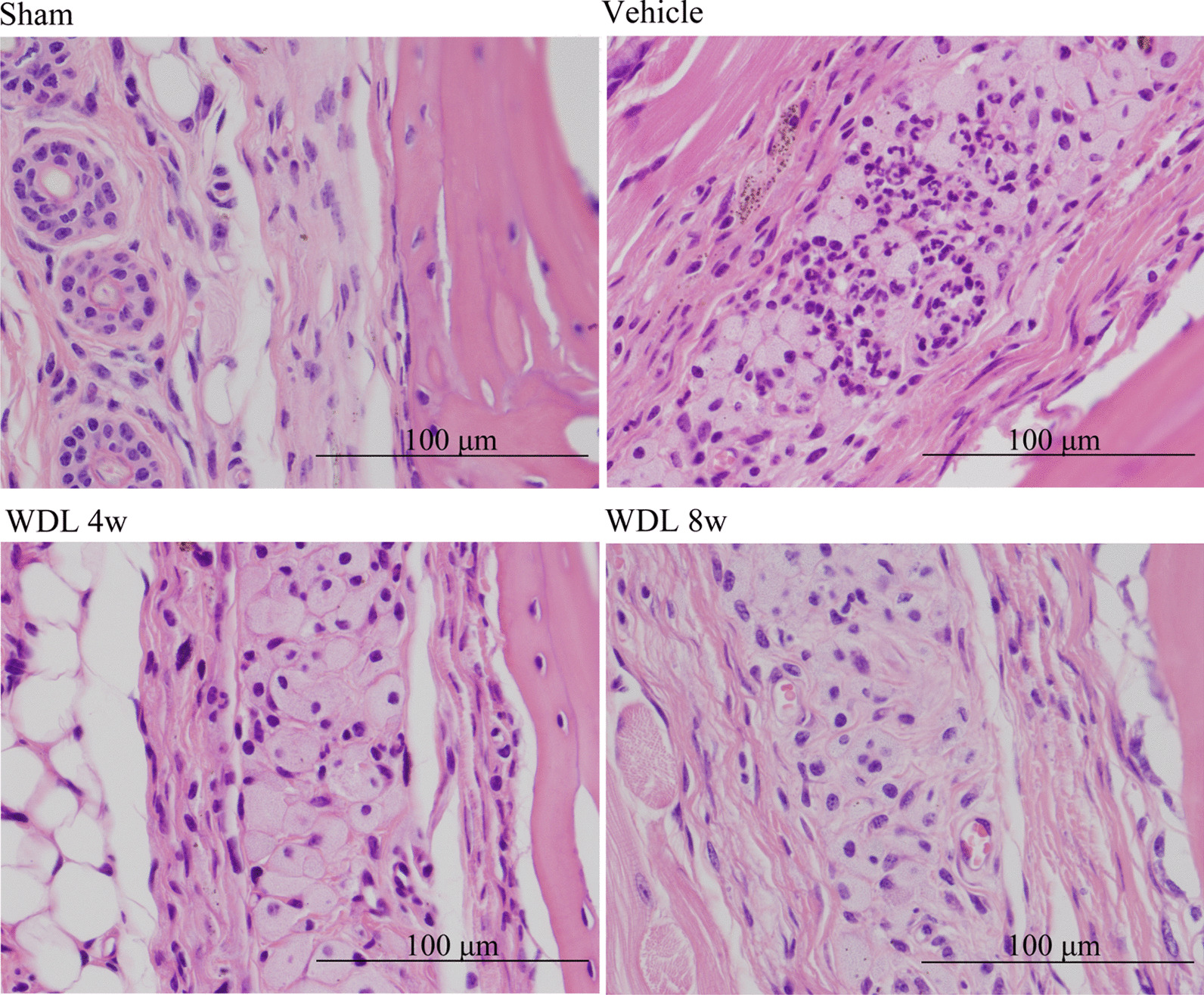
Hematoxylin and eosin (H&E) staining of periosteum in mice calvarial section. Multinucleated giant cells were observed in the groups injected with PS particles (Magnification: × 40; scale bar: 100 μm)

**Fig. 4:**
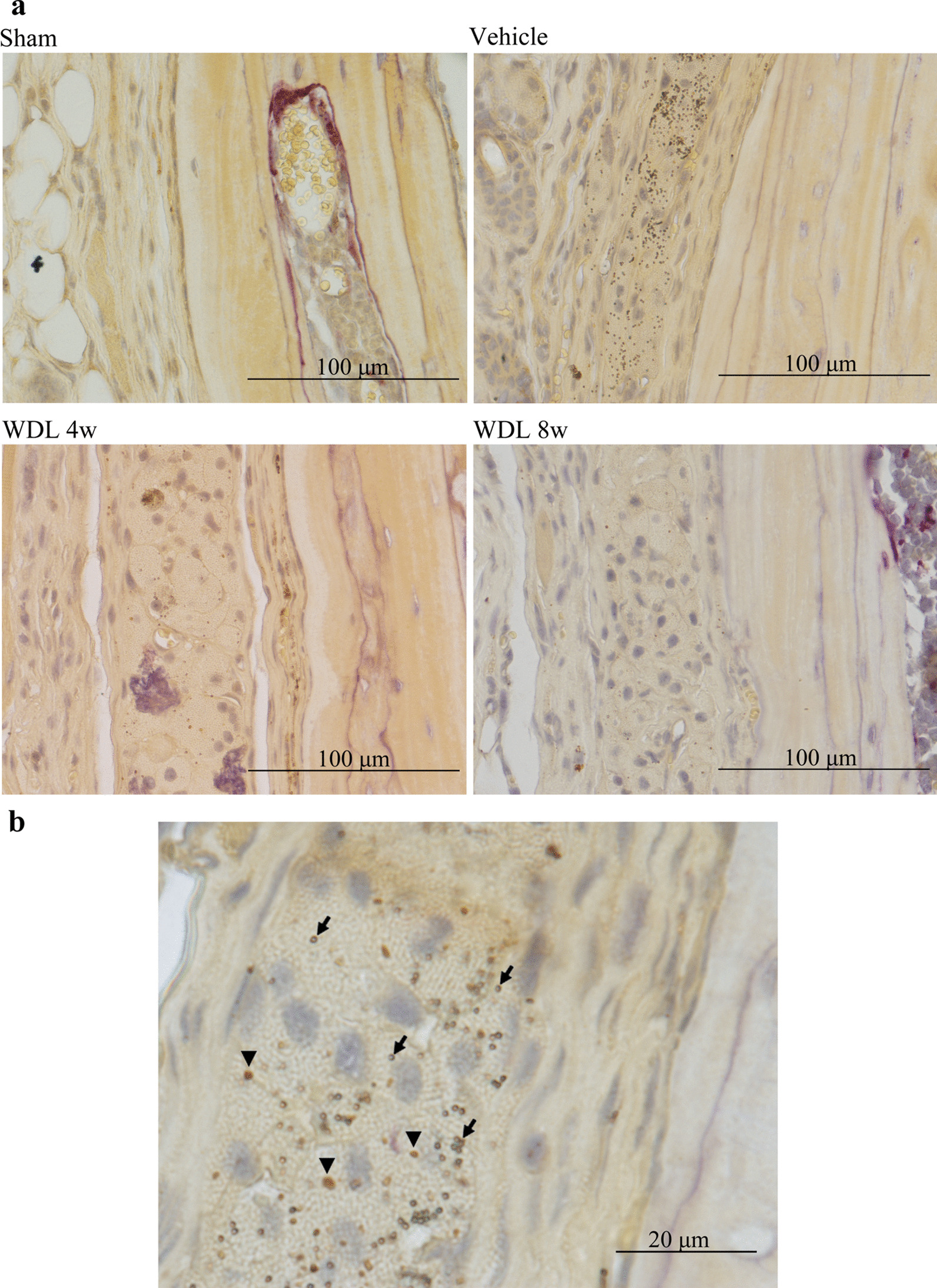
TRAP staining indicated that PS particles exist in the periosteal cells and multinucleated giant cells in mice calvarial tissue (**a**) (Magnification × 40 Scale bar: 100 μm). Different shapes of PS particles and melanin granules observed in TRAP staining. Arrow, PS particle; Arrow head, melanin granule (**b**) (Magnification × 100 Scale bar: 20 μm)

Histological analysis with H&E staining was used to evaluate the inflammatory response. Pseudomembrane proliferation occurred in the vehicle and WDL groups. The morphology of the cells in the periosteum was observed to change to a circle-shaped contour, while the cells resembled a flat contour in the sham group. Multinucleated giant cells were found in the surrounding periosteum (Fig. 3). TRAP staining was used to highlight polymer particles in the periosteal cells and multinucleated giant cells (Fig. 4a, b).

### Osteoclasts around the bone perimeter

**Fig. 5:**
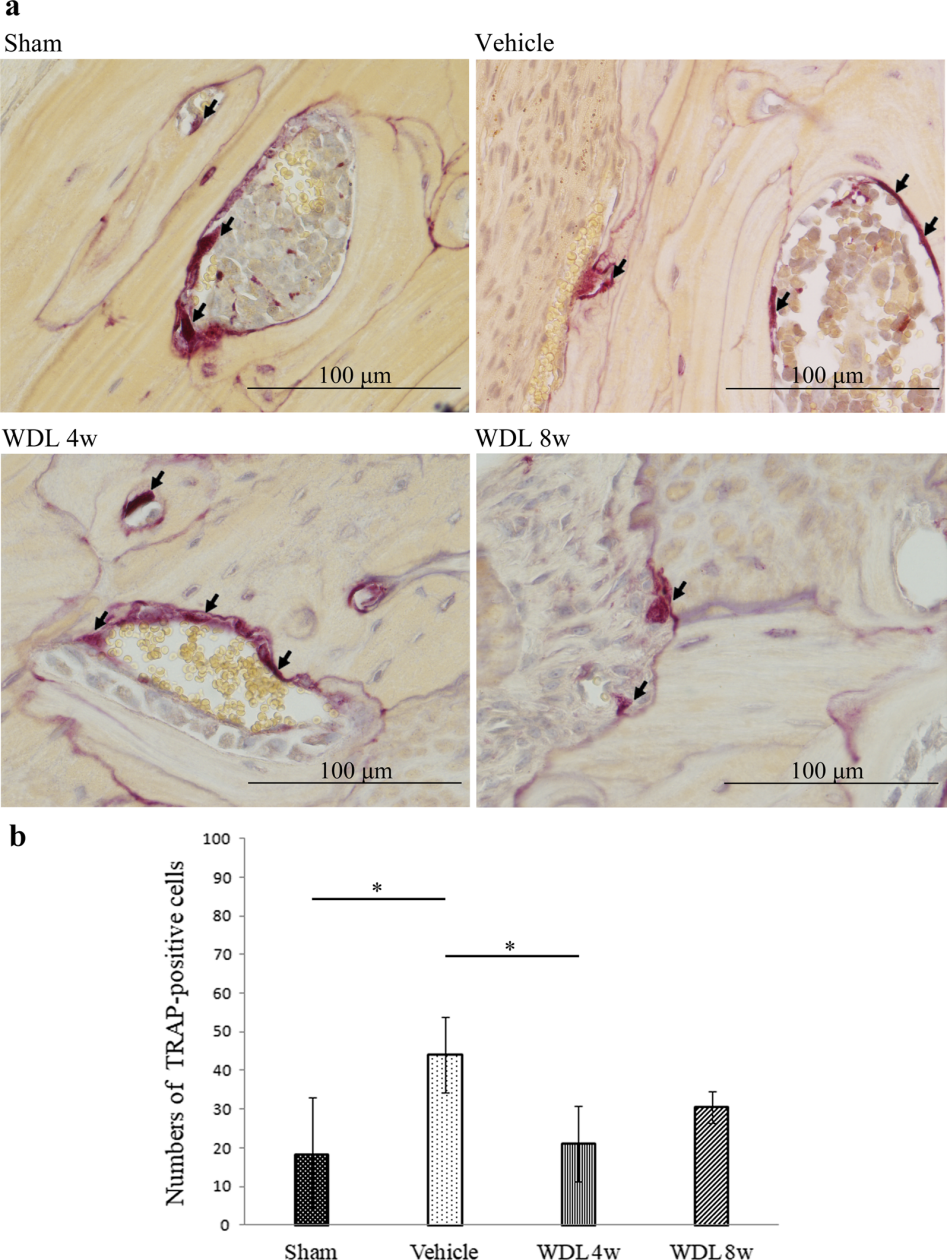
Typical samples from micro-CT with purple staining showing TRAP-positive osteoclasts (**a**) (Magnification: × 40; Scale bar: 100 μm). Average number of TRAP-positive cells from each group are presented as the mean ± SD (**b**) (*p < 0.05)

TRAP stain was used to highlight osteoclasts around the calvaria to calculate the osteoclast numbers in each group. The results showed the osteoclast numbers in the vehicle group increased significantly in comparison to the sham group (43.7 ± 10.1 in vehicle group versus 18.2 ± 14.5 in sham group, *p* < 0.05), demonstrating that the polymer particles likely induced osteolysis. Furthermore, there was a significant reduction in osteoclast numbers in the WDL 4w group in comparison to the vehicle group (21.1 ± 9.8 in WDL 4w group versus 43.7 ± 10.1 in vehicle group, *p* < 0.05). However, osteoclast number in WDL 8w group (30.6 ± 4.0) was no significantly different to the WDL 4w group (Fig. 5b).

## Discussion

Osteolysis is one of the major long-term complications affecting patients who undergo AJR. Chinese herbal medicine is generally considered to be milder than pharmaceutical treatments and does not produce strong adverse effects. This study aimed to investigate the potential use of the Chinese herbal medicine wedelolactone for reducing the incidence of particle-induced osteolysis.

Bisphosphonates (BPs) are commonly used to treat conditions of metabolic bone loss, such as osteoporosis [7], but have also been shown to inhibit particle-induced osteolysis [39]. However, the long-term use of BPs and other pharmaceuticals often results in serious adverse effects, such as an increased risk of osteonecrosis of the jaw, atypical femur fractures, atrial fibrillation, and esophageal cancer [6]. Statins, a drug usually used to lower blood cholesterol levels and reduce the risk of symptoms related to atherosclerosis, targets the mevalonate pathway of osteoclasts, which affect the same inhibition mechanism as bisphosphonates. Statins have been shown to markedly reduce the severity of particle-induced osteolysis in a murine calvarial model [5]. However, as with BPs, the use of statins can present a number of side effects when used long-term, such as rhabdomyolysis, cognitive loss, neuropathy, hepatic dysfunction, and sexual dysfunction [8].

There is increasing interest in alternative methods such as traditional Chinese medicine for treating diseases as clinicians look to reduce long-term complications associated with conventional medicine. It has reported that postmenopausal Chinese women with greater fruit intake have a significantly higher BMD than comparable women with a lower fruit intake [40]. Flavonoids, found in a wide diversity of food derived from fruit, have been recognized as potential dietary components to promote bone health [41, 42]. Nam et al. also indicated that traditional mixed extracts of medicinal herbs can effectively inhibit the expression of inflammatory mediators in gouty arthritis on monosodium urate (MSU) crystals-induced gouty inflammation, demonstrating its potential for treating gouty arthritis [43]. Phytoestrogens, which are natural compounds that act to maintain healthy bones, have been shown to protect against postmenopausal bone loss [20]. This protective mechanism has been demonstrated with flavones [44, 45], flavanones [46, 47], flavonols [48], coumestans [49], and triterpenoids [46, 50]. Some phytoestrogens also have the ability to reduce osteolysis by blocking some modules in the RANKL signaling pathway, and subsequently reducing the release of cytokines [44, 45, 47, 51].

To our best knowledge, no studies to date have investigated whether WDL can reduce the risk of particle-induced osteolysis using an *in-vivo* murine calvarial model. The concentration of WDL used in this study was adopted from Tsai et al. [21] who showed that a low oral dose of WDL (4 mg/kg) administered for 4 weeks significantly suppressed the growth of prostate cancer cells. The results of our study showed that the BMD was significantly greater in the WDL 8w group (0.784 ± 0.014 mg/cc) than in the vehicle group (0.744 ± 0.032 mg/cc) (Fig. 2b). However, there was no significant difference between the WDL 4w and 8w groups in terms of the mean values of BV/TV and Tb.Th. On the other hand, the osteoclast numbers were significantly lower in the WDL 4w group (21.1 ± 9.8) and WDL 8w group (30.6 ± 4.0) than the vehicle group (44.1 ± 9.8) (Fig. 5).

Multinucleated giant cells are typically generated after implantation of medical advices, artificial joint or biomaterials, taking the form of foreign body giant cells. The formation of these giant cells is the end-stage of the inflammation or wound healing response [52]. Osteoclasts are specialized multinuclear giant cells derived from monocyte/macrophage lineage cells [53]. In this study, multinucleated giant cells were observed in the groups injected with polymer particles (Fig. 3). In addition, TRAP staining indicated that polymer particles were present in the periosteal cells and multinucleated giant cells in the mouse calvarial tissue (Fig. 4a, b).

Wear particles have been found in surrounding tissue after implantation of many different materials used in TJR, including ultra-high molecular weight polyethylene (UHMWPE) [54], poly(methyl methacrylate) (PMMA) [54], ceramics [54], metallic CoCrMo [55] and titanium alloy [55, 56]. Some studies used titanium particles to create particle-induced osteolysis animal models [44, 46, 53, 57, 58]. However, when used in joint replacements, titanium or its alloys generate fewer wear particles than polymers because of the relatively low mechanical strength of polymeric materials. In the authors’ previous animal studies, UHMWPE wear debris-induced animal models were developed to analyze the in vivo biological response to highly cross-linked and vitamin E-stabilized polyethylene [10] and to evaluate the potential role of strontium ranelate-contained medicine to treat osteolysis [11]. However, considerable time was spent preparing enough wear debris for these animal models, while polystyrene (PS) particles are readily available and are widely used both commercially and for biomedical research [59, 60]. PS particles have been used in many animal studies to create particle-induced osteolysis animal models [36–38]. Furthermore, given that the size distribution and shape of PS particles are easier to control, this study used PS particles for the animal model.

Previous studies treated murine calvarial osteolysis with bioactive compounds for 10–14 days after implantation of foreign particles [12, 44, 45, 47, 50, 61]. For instance, icariin, a bioactive flavonoid, has been proven to inhibit postmenopausal osteoporosis. Shao et al. gavage-fed mice with icariin at doses of 0.1 mg/g and 0.3 mg/g for 14 days to examine the effects on osteolysis in a particle-induced murine calvarial model. The results showed an increase in BMD and BV/TV over the control model, and the number of TRAP positive cells decreased [12]. Similarly, ursolic acid is an abundant triterpenoid present in over one hundred species of plants. It has been reported that ursolic acid isolated from loquat leaves can reduce bone loss in OVX mice [46]. Jiang et al. treated mice with 10 mg/kg and 40 mg/kg doses of ursolic acid administered through intraperitoneal injections for 14 days and found that ursolic acid protects against wear particle-induced osteolysis by suppressing osteoclast formation and function [50]. The treatment period in this current study, 4 weeks and 8 weeks, was longer than the referenced studies which only treated for a short-term of 2 weeks. No adverse effects were observed in this study after treating the mice for 8 weeks with WDL.

Bone remodeling is a dynamic equilibrium with molecular mechanisms, such as RANK/RANKL/OPG [62], NF-κB [63], and Wnt/BMP (bone morphogenic protein) [57, 64] signaling pathways playing critical roles in osteolysis. Although the trigger mechanisms for osteolysis are not yet fully understood, it is known that one of the mechanisms is the receptor activation of NF-κB ligand (RANKL) and osteoprotegerin (OPG) secreted from osteoblasts and osteogenic stromal cells, both of which act to maintain a balance between bone generation and resorption [62]. RANKL is required for the differentiation of osteoclast precursors into mature osteoclasts [58]. As the ratio of RANKL/OPG increases, the osteoclast precursors are easier influenced by RANKL signaling through the downstream activation of NF-κB/c-fos/NFATc1, subsequently causing the precursors to differentiate into mature osteoclasts. On the other hand, macrophages also plays a key role in wear particle-induced osteolysis [63, 65–68]. Cytokines (TNF-α and IL-1β, etc.) and other mediators of pro-inflammation from activated macrophages can regulate or stimulate other tissue-resident macrophages to promote osteoclastogenesis [66]. These cytokines also regulate JNK and the p38/ERK signaling pathway to induce NFATc1, one of the downstream factors in the RANKL signaling pathway, which can lead to osteolysis.

Studies have shown that some compounds from Chinese herbal medicines can treat particle-induced osteolysis by inhibiting the modules in the NF-κB signaling pathway, the main mechanism in the regulation of osteolysis, to effect the balance of osteoclasts and osteoblasts [12, 45, 50, 61, 68]. WDL is known for its ability to block the phosphorylation of IκBα, which acts to regulate the transcription of NF-κB mediated genes, inhibiting LPS-induced pro-inflammation [69]. Annie et al. demonstrated how an extract from *Wedelia chinensis* attenuated OVX-induced bone loss in mice [70]. WDL extracted from *Ecliptae herba* has been shown to inhibit osteoclastogenesis of RAW 264.7 cells treated with RANKL [35], and prevent OVX-induced bone loss by inhibiting osteoclast activity and enhancing osteoblast activity [34]. Furthermore, it has been confirmed that WDL can regulate the RANKL-related NF-κB/c-fos/NFATc1 pathway to suppress osteoclastogenesis [26, 71], and also regulate the Wnt/β-catenin signaling pathway to induce osteoblastogenesis [71, 72]. The authors concluded that oral WDL could improve bone formation and inhibit resorption by affecting the balance of osteoclasts and osteoblasts. However, the mechanism leading to the inhibition of osteolysis by WDL still needs to be determined.

Some limitations of this study should be mentioned. First, murine calvarial models allow for a low-cost study with relatively quick results, but the models use a flat bone instead of a long bone and the particles are injected on the cortical bone surface rather than into cancellous bone. Second, as detailed above, the WDL dose used in this study was adopted from other related publications. However, the most effective dose for treating osteolysis in vivo has yet to be determined and requires further study. Third, osteoclast numbers were counted through qualitative analysis, not quantitative analysis. When injected onto the calvaria, the particles randomly precipitated and then a section was chosen for histological staining. This sampling approach may not represent true osteoclast numbers. Further studies are recommended to investigate inflammatory makers. Accepting the above limitations, this animal study identified the potential role of wedelolactone for treating particle-induced osteolysis.

## Conclusions

This study indicated that wedelolactone (WDL), a Chinese herbal medicine, could help to maintain bone quality. Oral WDL was shown to suppress osteoclast numbers and maintain the level of BMD over time in a particle-induced osteolysis murine clavarial model. Moving forward, WDL could be potentially developed as a functional food for lowering the risk of particle-induced osteolysis after total joint replacement.

## Data Availability

The data sets used and/or analyzed during the current study are available from the corresponding author on reasonable request.

## Abbreviations

TJR: Total Joint Replacement
WDL: Wedelolactone
BMD: Bone Mineral Density
BV/TV: Bone Volume/Tissue Volume
Tb.Th: Trabecular Thickness
ISO: International Organization for Standardization
RANKL: Receptor activator of nuclear factor kappa-Β ligand
OPG: Osteoprotegerin
OVX: Ovariectomy
